
JOURNAL INFORMATION
==============================
NLM Title Abbreviation: FEBS Open Bio
Journal ID: FEBS Open Bio
NLM Journal ID: 101580716
Journal ID: FEB4

FEBS Open Bio

EISSN: 2211-5463
Publisher: Wiley

ARTICLE INFORMATION
==============================
PMCID: PMC5302054
DOI: 10.1016/j.fob.2015.12.001
Article ID: FEB4S2211546315001072
Article version: 1
Subjects: Publisher's Note, Publisher's Notes

Reviewers’ acknowledgement

Publication date: 2015
Electronic publication date: 2015 Dec 12
Volume: 5
First page: 942
Last page: 944
Copyright: FEBS Open Bio 5 (2015) 2211-5463 ©2015 The Authors. Published by FEBS Press and John Wiley & Sons Ltd.
License: This is an open access article under the terms of the Creative Commons Attribution License, which permits use, distribution and reproduction in any medium, provided the original work is properly cited.
License URL: https://creativecommons.org/licenses/by/4.0/

Page count: 3
source-schema-version-number: 2.0
component-id: feb4s2211546315001072
cover-date: January 01, 2015
details-of-publishers-convertor: Converter:WILEY_ML3GV2_TO_NLMPMC version:5.0.6 mode:remove_FC converted:10.02.2017

(2015), Reviewers’ acknowledgement, FEBS Open Bio, 5, doi: 10.1016/j.fob.2015.12.001

==============================
FEBS Open Bio 5 (2015) 942–944

FEBS openbio journal homepage: www.elsevier.com/locate/febsopenbio

### Reviewers' acknowledgement

The Editors of *FEBS Open Bio* would like to thank all those who have given their time and expertise to review articles submitted for publication in Volume 5. Although *FEBS Open Bio* does not seek to judge the importance of submissions, our reviewers carefully scrutinize the experimental design and results of all papers, and challenge authors about their conclusions.

The names of these reviewers are listed below; we apologize if we have inadvertently omitted anyone.

Gaber Abogadallah
Mohammed Akaaboune
Hidetaka Akita
Vivek Anantharaman
Asaithamby Aroumougame
Hiroshi Asahara
Sassan Asgari
Massimo Aureli
Claudia Bagni
Keith D Baker
Mitchell F Balish
Manju Bansal
Marie-Anne Barny
Nidhi Batra
Michael Baumann
Boon Huat Bay
Bhaskar Bhattacharya
Alexander Bird
Gisele Bonne
Uwe Theo Bornscheuer
David Bowen
Stephanie Marie Bozonet
Eun Kyoung Yim Breuer
Dennis Christopher Bruemmer
Pawel Burkhardt
Peter Burkovics
Arsène Burny
Chris Bystroff
Antoni Camins
Viviana Caputo
Giuseppina Caretti
Anthony Carruthers
Walee Chamulitrat
Vinod Chandra
Hsueh-Wei Chang
Yubing Chen
N Cheng
Yu-Ching Cheng
Fulvio Chiacchiera
Hitoshi Chiba
Evangelia Chrysina
Jared C Cochran
Matteo Coen
William B Coleman
Bryan Copple
Maria Almira Correia
Jeroen Corver
Gael Cristofari
Ixchelt Cuaranta-Monroy
Yongchun Cui
Eoin Cummins
Francesca Cutruzzola
Douglas Darling
Swapan Das
Benjamin Davies
Julia M Davies
Elena De Falco
Bert L De Groot
Albert de la Chapelle
Vito De Pinto
Shraddha Desai
Yvan Devaux
Simona Gabriella Di Santo
Jeffrey Dick
Ning Ding
Peter Dodds
Harsh Dweep
Ruth Y Eberhardt
Sabrina Ehnert
Felix Elortza
Ignacio José Encío
Astrid M Engel
Péter Erdélyi
Evangelos Evangelou
Elena Evguenieva-Hackenberg
Julián M Fernández
Carlos Fitzsimons
Ervin Fodor
Yanfang Fu
Tomokazu Fukuda
Tsutomu Fukuwatari
Haichun Gao
Jianwei Gao
Ivan Garcia-Bassets
Cesare Gargioli
James Peter Garnett
Anne-Claude Gavin
Pedro M Geraldes
Jonathan M Gibbins
Elen Gócza
Maurice Godfrey
Erwin Goldberg
Pierre Golstein
Renata Goncalves
Herminia González-Navarro
Radhika Gopal
Wendy Gordon
Adrian E Granada
Michal Grzybek
Rong Guan
Andre Guillouzo
Erich Gulbins
Longgen Guo
Artur Gurgul
Volker Gurtler
José María Gutiérrez
Krzysztof Guzik
George Hajishengallis
Carmen Verissima Ferreira Halder
Christiane Hammerschmidt-Kamper
Chunsheng Han
Bassim Hassan
Graham Hatfull
Fei He
Jianxing He
Lizhi He
Paul W Hill
Klaus H Hoffmann
Leif G Hommers
Stefan Hörtensteiner
Jinjun Hou
Guy A Howard
Michael T Howell
Huey W Huang
Jianhua Huang
Stephen P Hunt
Wilhelmina Huston
Perpetua Uchechi Ibekwe
Masaaki Ikeda
George Jacoby
Michael Janitz
Isabelle Jariel-Encontre
Hans E Jörnvall
Jihang Ju
JW Jukema http://dx.doi.org/10.1016/j.fob.2015.12.001

Alexander Jurko
Ravi C Kalathur
Lynn Kamerlin
Frederick WK Kan
Dongchul Kang
Tomasz Kantyka
Ibrahim Kassis
Takenobu Katagiri
Hidekazu Katayama
John Katsaras
Mark Kester
Hippokratis D Kiaris
Hiroshi Kimura
Stefan Kins
Diana Kirilovsky
André Kleinridders
Saulius Klimašauskas
Alex S Knisely
Takeshi Kobayashi
Karel Koberna
Warren Kruger
Satoshi Kubota
Sandeep Kumar
Minliang Kuo
Manabu Kurokawa
Tricia L Larose
Justin Durla Lathia
Gennaro Mariano Lenato
Adam Lesner
Darren P Letley
Jianrong Li
Liande Li
Xing Li
Zhaofeng Li
Shuang Liang
Krzysztof Liberek
Aifu Lin
Dan B Lindholm
Baogang Liu
Leyuan Liu
Xiang Liu
Kent Lloyd
Gloria Lopez-Castejon
Marina Lotti
Giuseppe Lucarelli
Yonglun Luo
Yongjie Ma
Marco A Magalhaes
Pawel Mak
Bettina Malnic
Tszkwong Man
Loren J Martin
Isabel Marzo
Nikolas P Mastroyiannopoulos
Peter Mayser
David McClay
Stephen McPherson
Milagros Medina
Chris Meisinger
Qinglai Meng
Qingyong Meng
Martin Michaelis
Gracjan Michlewski
Todd Miller
Sarah Milton
Ismael Mingarro
Hamed Mirzaei-Souderjani
David Mitchell
Abhisek Mitra
Hiroyuki Mizoguchi
Nadia Monesi
Tony Moore
Marcelo Mori
Simon Moxon
Sourav Mukherjee
Conrad W Mullineaux
Maasaki Muramatsu
Noriyuki Nagahara
Hideaki Nagase
Eiko Nakamaru-Ogiso
Yoshimasa Nakamura
Masayuki Nara
Joseph Ndisang
Daniel Robert Neill
Jochen Neuhaus
Dietbert Neumann
Linda Nicholson
Noa Noy
David O'Callaghan
Rodney P O'Connor
David Ochoa
Karen A Ocorr
Brett Olds
Carla Oliveira
Eric A Ortlund
Amanda Padovan
Carlos M Palmeira
Ann C Palmenberg
Jae Kweon Park
Kyungho Park
Suekyung Park
Laurence Parnell
Carrie Partch
Jacques M Pasteels
Luciana Paulino
Chanyarat Paungfoo-Lonhienne
Ludmila Pawlikowska
Jose Carlos Perales
Jose E Perez-Ortin
Michael A Persinger
John Phillips
Robert K Poole
Joanna Poulton
Maureen A Powers
Rolf A Prade
Alessandro Prinetti
Maurizio Provenzano
Kefeng Qin
Peter J Quinn
Premraj Rajkumar
Om Rajora
Sonia Ramos
Ajay Rana
Didier Raoult
Neil David Rawlings
Charles S Redwood
James Reecy
Peter J Reiser
Ricardo M Richardson
Cesar Rodriguez
T Roeder
Ana Rojas
Ioulia Rouzina
Andrew Rowley
Michael John Russell
Jean-Marie Ruysschaert
Simone Sabbioneda
Marat R Sadykov
Naoto Sakamoto
Prasanna Kumar Santhekadur
Rajkumar Savai
Deborah Schechtman
Santiago Schnell
David A Schooley
Stefan Schülke
Radislav Sedlacek
Sergey A Selkov
Gerhard Friedrich Sengle
Tanima Sengupta
Nicolas Sergeant
Gaetano Serviddio
Yechiel Shai
Ravi Shankar
Sudha Sharma
Zhigang She
Wei Shen
Seiji Shiota
Amit Singh
Suren Singh
David Smith
Chumporn Soowannayan
Maria João Sousa
David Stefan
LKM Steinbusch
George P Studzinski
Carmelo Sturiale
Kartik Subramanian
Suresh Sudarsan
Fumihiro Sugiyama
Jie Sun
Zheng Sun
Toru Taguchi
Nobuyuki Takahashi
Isei Tanida
Utpal Tatu
Philip Tedbury
Robert A Terkeltaub
Fuminori Tokunaga
Phong Tran
Prateek Tripathi
Andrés Trostchansky
Kuen-Jer Tsai
Rodney K Tweten
Yoshikazu Uchida
Shinichi Ueno
Willem JH van Berkel
Andras Varadi
Gabriele Varani
Mariángela Vargas
Vitor Manuel de Oliveira Vasconcelos
A Villunger
Amarjit S Virdi
Rui Vitorino
Vladana Vukojevic
Malte Wachsmuth

Sarah Walmsley
Guannan Wang
Hao Wang
Jian Wang
Liya Wang
Shukui Wang
Yong Wang
Yuanzhong Wang
Craig Warden
Wen Weihong
Ervin Welker
Mick M Welling
Nicolas Werbeck
Claudio Chrysostomo Werneck
Thomas Winckler
Thorsten Wohland
David W Wood
David C Wright
Meng Wu
Ming Xu
Zhongping Xu
Zhihong Xue
Masayuki Yamamoto
Soichiro Yamamura
Naohide Yamashita
Xialong Yang
Lin Ye
Xueren Yin
Fang Yuan
Shaochun Yuan
Haiyan Zhang
Jinyuan Zhang
Jinzhong Zhang
Mingliang Zhang
Qiang Zhang
Chunxia Zhao
Qingguo Zhao
Yongchao Zhao
